# DEAD-Box Helicases: Sensors, Regulators, and Effectors for Antiviral Defense

**DOI:** 10.3390/v12020181

**Published:** 2020-02-05

**Authors:** Frances Taschuk, Sara Cherry

**Affiliations:** Department of Microbiology, University of Pennsylvania, 3450 Hamilton Walk, Philadelphia, PA 19104, USA; ftaschuk@pennmedicine.upenn.edu

**Keywords:** RNA-protein interactions, intrinsic immunity, antiviral, virus-host interactions, RNA helicase, DEAD-box helicase, viral replication, ribonucleoprotein complexes

## Abstract

DEAD-box helicases are a large family of conserved RNA-binding proteins that belong to the broader group of cellular DExD/H helicases. Members of the DEAD-box helicase family have roles throughout cellular RNA metabolism from biogenesis to decay. Moreover, there is emerging evidence that cellular RNA helicases, including DEAD-box helicases, play roles in the recognition of foreign nucleic acids and the modulation of viral infection. As intracellular parasites, viruses must evade detection by innate immune sensing mechanisms and degradation by cellular machinery while also manipulating host cell processes to facilitate replication. The ability of DEAD-box helicases to recognize RNA in a sequence-independent manner, as well as the breadth of cellular functions carried out by members of this family, lead them to influence innate recognition and viral infections in multiple ways. Indeed, DEAD-box helicases have been shown to contribute to intracellular immune sensing, act as antiviral effectors, and even to be coopted by viruses to promote their replication. However, our understanding of the mechanisms underlying these interactions, as well as the cellular roles of DEAD-box helicases themselves, is limited in many cases. We will discuss the diverse roles that members of the DEAD-box helicase family play during viral infections.

## 1. The DEAD-box Helicase Family of RNA-Binding Proteins

The broad family of DExD/H helicases, including DEAD-box helicases and the related DEAH-box helicases, belongs to superfamily 2 of the RNA helicase family. This family is characterized by a series of amino acid motifs that form the RNA and ATP binding sites of the helicase core [1,2]; DEAD-box helicases contain the amino acid sequence DEAD in motif II as well as an additional upstream Q motif [3]. DEAD-box helicases are conserved from bacteria to mammals [4]. Structurally, the DEAD-box helicase core contains two domains resembling bacterial RecA, which cooperatively bind RNA and ATP, interacting with RNA along the sugar-phosphate backbone [4]. Because of the lack of interactions with the RNA nucleotide bases, the binding of DEAD-box helicases to RNA is thought to be generally sequence-independent but structure-dependent [5,6].

In vitro studies of DEAD-box helicase unwinding activity have revealed ATP-dependent RNA helicase function, but these proteins tend to have only weakly processive helicase activity, and instead are thought to promote local RNA:RNA rearrangements [7]. Beyond their activity on RNA duplexes, studies have also revealed that some members of this family have other activities regulating nucleic acid binding, including the modulation of RNA:DNA interactions, DNA:DNA interactions, and RNA-protein (RNP) complex remodeling [8,9].

Although DEAD-box helicases share a conserved core domain, variable N- and C-terminal regions allow members of this protein family to act on diverse targets. These flanking domains target the helicases to particular RNAs or proteins; the diversity of these regions accounts for the breadth of function observed within this protein family [7]. As a result of their diversity of binding and function, DEAD-box RNA helicases are involved in all aspects of cellular RNA metabolism, including transcription, splicing, microRNA biogenesis, ribosomal RNA processing, RNA export, translation, RNP granule formation, and decay [10]. Moreover, they are also involved in cellular stress responses, contributing to double-strand break (DSB) repair [11], stress granule formation, and antimicrobial responses [12].

## 2. DEAD-Box Helicases in Canonical Innate Immune Sensing

Innate immune defenses depend upon the recognition of invading pathogens by the host, and viral infections, including RNA viruses, are largely sensed through nucleic acid recognition. RNA viruses present a host cell with various foreign RNA features, such as unique ends, structured RNA elements, or dsRNA replication intermediates, which can be recognized as hallmarks of infection. RNA binding proteins thus play important roles in the sensing of viral infection, and DExD/H helicases are well-suited to recognize structural features characteristic of viral RNAs. 

**Figure 1:**
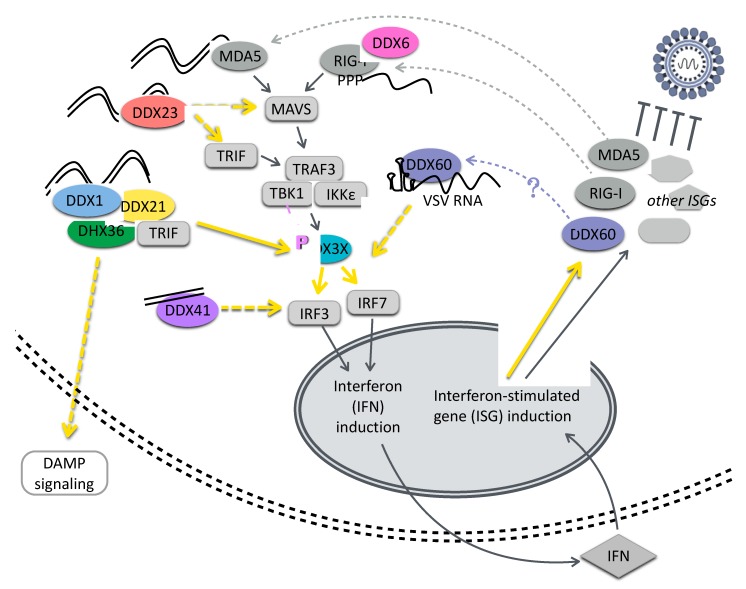
DEAD-box helicases contribute to innate immune signaling. DEAD-box helicases have been demonstrated to serve as sensors for nucleic acids, including dsRNA, cytoplasmic DNA, and viral RNAs, leading to the induction of interferon and interferon-stimulated genes. DEAD-box helicases also contribute to innate immune signaling downstream of nucleic acid sensing by mediating protein–protein interactions and promoting DAMP signaling. Moreover, some DEAD-box helicases are ISGs, suggesting positive feedback on their innate immune roles. RIG-I and MDA-5, while considered members of the RIG-I-like receptors (RLR) family, contain related DExD/H-box RNA binding domains. DEAD-box helicases are shown in color and other cellular components are shown in gray. Dotted yellow lines are used where there may be intermediate steps in the indicated relationship. (DAMP, damage-associated molecular pattern; IFN, interferon; ISG, interferon-stimulated gene; TLR, toll-like receptor; VSV, vesicular stomatitis virus).

The canonical cytoplasmic sensors of viral RNA, the RIG-I-like receptors (RLRs) RIG-I and MDA-5, contain DExD/H-box helicase domains [13]. RIG-I recognizes uncapped viral RNAsand short dsRNAs containing a 5′ triphosphate, while MDA-5 binds longer dsRNA replication intermediates [13,14,15]. In addition to the helicase core domain, these proteins contain caspase activation and recruitment domains (CARDs).RNA recognition by these RLRs leads to oligomerization of CARDs and activation of antiviral signaling complexes [16,17]. RIG-I and MDA5 both signal through the CARD-domain-containing adapter protein MAVS, causing the polymerization and activation of MAVS on the mitochondrial membrane [14]. This leads to the activation of IRF3 and IRF7, promoting the transcription of IFN-β [14]. Type I interferons subsequently induce the transcription of hundreds of interferon-stimulated genes (ISGs) that control infection, including a number of RNA helicases (Figure 1).

DEAD-box helicases have been shown to contribute to cytosolic viral RNA recognition and to feed into these canonical immune signaling pathways, sometimes acting in concert with DEAH-box helicases. Several DEAD-box helicases promote interferon induction or other inflammatory signaling. DDX1 contributes to IFN-β activation during transmissible gastroenteritis virus infection via its interaction with the nonstructural protein nsP14 [18] and interacts with RelA potentiating NF-κB-dependent transcription, a function which requires intact helicase activity [19]. DDX21 is antiviral against dengue virus, promoting IFN-β induction during infection; in turn, the dengue virus ns2B/3 protein complex directs the proteasomal degradation of DDX21 to counteract this signaling [20]. A complex of the helicases DDX1, DDX21, and DHX36 interacts with the adaptor protein TRIF and cytosolic dsRNA, leading to a TLR3-independent activation of IRF3/7 and interferon induction, thus suggesting an additional role for TRIF beyond its known functions in toll-like receptor (TLR) signaling and endosomal sensing [21]. DDX21 has been further implicated in inflammatory signaling via the production of endogenous damage-associated molecular patterns (DAMPs). During influenza virus infection, DDX21 and TRIF are required for the secretion of the TLR DAMP ligand S100A9, which serves as a DAMP signal and results in the rapid production of inflammatory cytokines, including TNF and IL-6 [22].

Cytosolic DNA recognition represents another important surveillance mechanism for stimulating innate immune pathways in response to invasion by DNA viruses and bacteria, as the cytoplasm has a dearth of DNA under normal conditions. Much of this signaling is now known to occur via the cGAS-STING pathway, where DNA recognition by cGAS leads to the production of the cyclic dinucleotide cGAMP, which signals through STING, leading to phosphorylation of IRF3 by TBK1 [23,24]. However, DEAD-box helicases can also contribute to dsDNA sensing: DDX41 senses dsDNA from diverse intracellular pathogens, including bacteria and DNA viruses, and upregulates interferon and cytokine responses [25]. DDX41 has been shown to bind DNA via the walker A and B motifs of its helicase domain, to interact with STING, and to be required for the activation of TBK1 [25].

While the innate immune roles of some DEAD-box proteins involve nucleic acid sensing, other innate immune functions involve protein–protein interactions with DEAD-box-helicases and are nucleic acid independent. DDX3X may serve as a scaffold for protein–protein interactions to promote the transduction of innate immune signaling cascades. The binding and phosphorylation of DDX3X by TBK1 facilitates IFN-β induction in response to diverse stimuli including poly(I:C), poly(dA:dT), and *Listeria monocytogenes* infection [26]. DDX3X also interacts with IKKε to mediate the phosphorylation of IRF3, and with IKKα to support its activation downstream of TLR7 [27,28].

Some DEAD-box helicases are themselves ISGs, further reinforcing their antiviral activity via positive feedback. RIG-I, MDA5, DDX60, and DDX60L are canonical ISGs [29,30,31]. DDX60 and DDX60L share approximately 70% amino acid identity and both have antiviral functions; however, their roles are distinct [29,31]. DDX60 binds vesicular stomatitis virus (VSV) ssRNA and dsRNA in vitro and promotes RLR activation [31]. DDX60 also contributes to RIG-I-independent degradation of HCV RNA, although its role in this process is unclear [32]. Activation of the EGF growth factor receptor attenuates DDX60 activity, so viruses known to activate EGF including influenza A virus, HCV, and VSV may be able to counteract DDX60 functions [32]. A study contrasting the responses of hepatocyte cell lines to IFN-γ uncovered the importance of DDX60L expression for controlling HCV infection, and ectopic expression of DDX60L was also found to restrict HCV infection independent of interferon signaling, suggesting an additional direct effector mechanism [29].

### 2.1. Negative Regulation of Innate Immune Responses by DEAD-Box Helicases and Viral Antagonism

Some DEAD-box helicase family members are negative regulators of interferon responses. This can occur via competition for RNA substrates or protein–protein interactions. For example, DDX24 binds dsRNA and ssRNA and sequesters it away from RLRs, thus interfering with IRF7 activation [33].

In the virus–host arms race, viruses have evolved diverse countermeasures to evade detection by the host and evade host immune responses. The existence of such countermeasures targeting DEAD-box helicase proteins further underscores these proteins’ importance as antiviral factors. A variety of viruses hijack or target DDX3X to promote infection. The binding of DDX3X by the vaccinia virus K7 protein interferes with DDX3X-dependent TBK1/IKKε activation [34]. Moreover, DDX3X binding to hepatitis B virus (HBV) polymerase also interferes with DDX3X-dependent TBK1/IKKε activation early in infection, while at later stages the helicase function of DDX3X is required to limit HBV transcription [35,36]. Hepatitis C virus (HCV) takes advantage of the immune modulatory function of DDX3X to support its own replication: the binding of DDX3X to the HCV 3′UTR activates IKKα-dependent noncanonical NF-κB transcription, promoting lipogenesis, which aids in viral replication [37]. However, DDX3X negatively regulates type I interferon production during arenavirus infection, and interactions between DDX3X and arenavirus nucleoprotein promote viral replication [38]. 

The relocalization of nuclear DDX21 to the cytoplasm during dengue virus infection contributes to interferon responses; this is consistent with previous reports of DDX21 involvement in innate immune sensing [20,21,22]. In turn, DENV NS2B/3 protease counteracts DDX21, leading to its proteasome-dependent degradation [20].

### 2.2. Conserved Interferon-Independent Antiviral Functions of DEAD-Box Helicases

**Figure 2:**
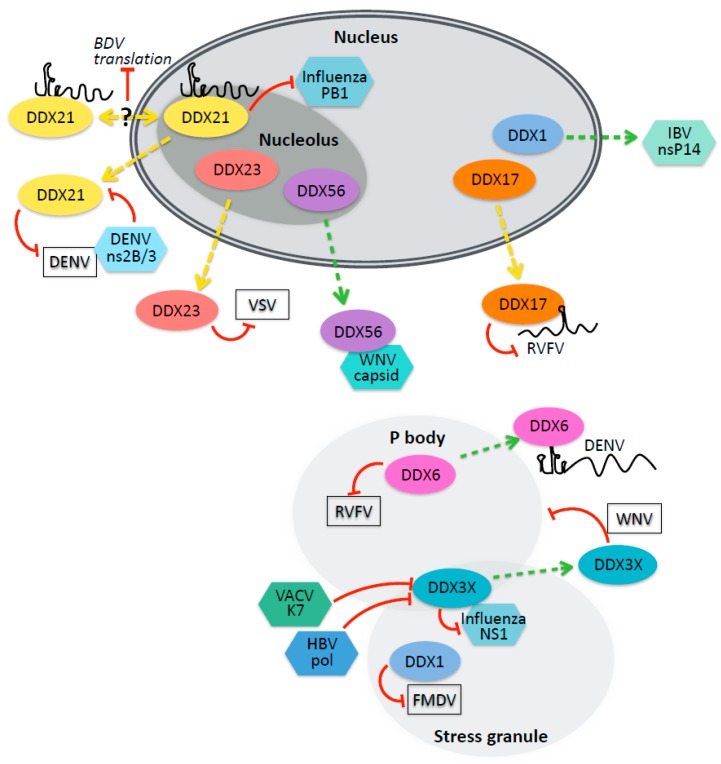
DEAD-box helicases with diverse cellular roles and localization interact with viral components and influence infection. DEAD-box helicases residing in nuclear, p-body, and stress granule compartments, have been implicated in viral infection. Interactions involve viral proteins, viral RNAs, or remain undefined. DEAD-box helicases can have positive or negative effects on infection. In some cases, viruses have been shown to counteract antiviral helicases, to alter the localization of helicases, or to disrupt RNA-protein (RNP) complexes. The red lines indicate negative interactions; the green arrows indicate positive interactions; and the dotted lines indicate protein relocalization. (VSV, vesicular stomatitis virus; DENV, dengue virus; RVFV, Rift Valley fever virus; WNV, West Nile virus; BDV, Borna disease virus; IBV, infections bronchitis virus, FMDV, foot and mouth disease virus; HBV, hepatitis B virus; VACV, vaccinia virus).

DEAD-box helicases can also contribute to antiviral effector functions independent of interferon signaling (Figure 2) [39]. Studies in invertebrates and plants have allowed the identification of several antiviral helicases which are necessarily interferon-independent, as these taxa lack an interferon system. Moreover, many of these antiviral functions are conserved in mammals. The screening of DEAD-box helicases in a *Drosophila* cell culture model of arthropod-borne RNA virus infection has revealed antiviral functions for several DEAD-box helicases that also have human homologs, including DDX6, DDX17, DDX24, and DDX56 [40,41].

DDX17 and DDX5 are nuclear resident paralogs that have diverse functions in transcription, splicing, miRNA biogenesis, mRNA export, and ribosome biogenesis [42]. DDX17 but not DDX5 was found to be antiviral against Rift Valley Fever Virus (RVFV). Moreover, DDX17 but not DDX5 relocalized to the cytoplasm during RVFV infection [41]. DDX17 activity against RVFV depends on the binding of a stem-loop structure onto the small segment of the tripartite RNA genome, and the addition of that stem-loop region to an unrelated virus rendered the chimeric virus sensitive to DDX17′s antiviral effect [41]. DDX17 may also have an antiviral function as a cofactor for the zinc-finger antiviral protein (ZAP), unwinding ZAP-bound viral mRNA to promote viral RNA decay by the RNA exosome [43,44]. The *Arabidopsis* homolog of DDX17, RH30, has also been shown to be antiviral against tombus viruses such as tomato bushy stunt virus and cucumber necrosis virus, both in plant hosts and a yeast surrogate host model [45]. RH30 relocalizes from the nucleus to the sites of viral replication and interacts with both viral proteins and viral RNA, particularly with structured cis-acting elements, thus interfering with template recruitment to the replicase complex [45]. In contrast to the antiviral roles of DDX17, knockdown of its paralog DDX5 attenuates replication of Japanese encephalitis virus (JEV), and helicase activity of DDX5 is required for this function [46]. DDX5 is recruited to the replication sites of JEV in the cytoplasm, and binds to the viral 3′ UTR to promote infection [46]. DDX3 is also recruited to JEV replication foci and directly promotes JEV replication by binding the untranslated regions [47].

DDX23 is a nuclear resident helicase with known roles in splicing. Studies in the invertebrate chordate amphioxus identified DDX23 as a dsRNA binding protein, and found that dsRNA binding was conserved in human cells [48]. How DDX23 controls viral infection in amphioxus is unclear, and whether DDX23 binding directly to viral RNA also impacts infection has not been explored. In humans, DDX23 binding to dsRNA potentiates antiviral signaling downstream of TRIF and MAVS [48]. Moreover, after dsRNA treatment or VSV infection, DDX23 translocates from the nucleus to the cytoplasm, suggesting that the relocalization of DDXs may be a common strategy to repurpose these RNA binding proteins for antiviral defense [48].

## 3. Cell Biological Interfaces of DEAD-Box Helicase Activity and Viral Infections

### 3.1. Nucleolar Helicases and Viral Infection

The nucleolus is the site of ribosomal RNA (rRNA) transcription, rRNA biogenesis, and assembly of ribosomal subunits. Nucleoli form as sites of concentrated processing factors around clusters of rDNA genes, and liquid–liquid phase separation drives the organization of subcompartments in which rRNA is cleaved and modified [49,50]. Many DEAD-box helicases localize to the nucleolus and participate in ribosome biogenesis; their precise functions are generally poorly understood, but they are thought to be involved in the remodeling of pre-ribosomal RNP complexes to promote rRNA biogenesis and in the release of snoRNAs after rRNA modification [51].

There is a growing appreciation for the role of the nucleolus in cellular stress responses, including responses to viral infections [49,52]. Known roles for nucleolar proteins in viral infection can involve interactions with RNA or proteins of nuclear-replicating viruses, the relocalization of nucleolar proteins to other cellular compartments, or targeting by viral proteins that localize to the nucleolus. For example, the nucleolar helicase DDX56, which normally functions in rRNA biogenesis [53], promotes West Nile virus infection [54,55,56]. The West Nile virus capsid biochemically interacts with DDX56 during infection, both in nucleoli and in the cytoplasm, and DDX56 helicase activity is required to promote the packaging of viral RNA into particles [54,55,56]. In the case of HIV-1, a DDX56–Gag interaction facilitates HIV-1 particle assembly, suggesting a broader role for DDX56 in viral assembly [57].

DDX21 is another nucleolar helicase with cellular functions in ribosomal RNA production [58] that also has a variety of moonlighting functions. DDX21 has been shown to be antiviral against both nuclear- and cytoplasmic-replicating RNA viruses through a variety of mechanisms. In the nucleus, DDX21 interacts with the Borna disease virus (BDV) RNA, binding the 5′ UTR of the BDV X/P mRNA and decreasing its translation [59]. In vitro RNA folding assays suggested that DDX21 binding causes structural alterations in the 5′UTR of the viral mRNA, thus interfering with the reinitiation of translation by ribosomes on this polycistronic message [59]. Influenza also replicates in the nucleus, but the activity of DDX21 against influenza appears not to require nucleic acid binding [60]; instead, a protein–protein interaction between DDX21 and the influenza PB1 polymerase inhibits the assembly of the viral replicase complex until being disrupted later in infection by increasing levels of the influenza NS1 protein [60]. During infection with DENV, DDX21 relocates to the cytoplasm and promotes interferon responses, as described above [20].

Taken together, these examples paint a picture where nucleolar proteins are influenced by, and exert effects on, cytoplasmic processes.

### 3.2. P-Body and Stress Granule Helicases in Viral Infection

P bodies are nonmembranous organelles in the cytosol that contribute to the regulation of cellular RNAs—resident RNAs can be stored, translationally repressed, and/or degraded [61]. Many viruses interface with P bodies in diverse ways (Reviewed in [62]). DDX6 resides in P bodies and facilitates P body assembly, as well as promoting the decapping and turnover of cellular mRNAs [63]. DDX6 also has cellular roles in the regulation of gene expression via translational repression [64].

DDX6 has been shown to have pro- or anti-viral roles in a variety of viral infections, including several arthropod-borne viruses. DDX6 enhances RIG-I signaling, interacting with both RIG-I and influenza viral RNA in infected cells [65]. However, DDX6 was also identified in a screen for suppressors of aberrant ISG activity; the deletion of DDX6 activates innate immune signaling pathways and primes cells to be more responsive to IFN, likely via the disruption of cytoplasmic RNA turnover and the accumulation of self RNA in P bodies [66]. Bunyaviruses snatch mRNA caps from host transcripts in P bodies, and thus cellular factors such as DDX6, which promote the decapping of host mRNAs and thereby deplete the pool of capped RNAs in P bodies, attenuate bunyaviral infection including La Crosse virus (LACV) and RVFV [40]. In mosquitoes, DDX6 is antiviral against the flaviviruses West Nile virus and Zika virus and is counteracted by the noncoding sfRNA derived from the flavivirus 3′UTR, which binds to DDX6 and sequesters it [67]. Human DDX6 is similarly antiviral against ZIKV and is sequestered by sfRNA [68].

Viruses can also hijack P-body components to aid in replication. DDX6 and other P body components have been found by mass spectrometry to bind the dengue virus 3′ UTR RNA, but in constrast to other flaviviruses, DENV infection is attenuated by DDX6 knockdown [69]. West Nile virus also subverts P body function by disrupting P bodies and recruiting component proteins including LSM1, GW182, DDX3, and XRN1 to viral replication sites where they positively contribute to replication by an unknown mechanism [70].

Stress granules are another type of nonmembranous organelle which form when translation is impaired [71]. The stress granule resident helicase DDX3X has diverse roles in interferon signaling (described in other sections of this review) as well as in other aspects of cell biology. DDX3X is antiviral against influenza A virus infection via its role in nucleating stress granules: the C-terminal domain interacts with influenza NS1 to sequester the viral protein in stress granules and limit the amount available to carry out viral replication [72]. It is also possible that many of the other roles of DDX3X in interferon signaling and viral replication are taking place in stress granules; however, this has not been explored. In plants and yeast, the helicases RH20/Ded1p (DDX3) are coopted to form part of the tombus virus replicase complex, and their presence helps to maintain full-length genome integrity and prevent recombination [73]. DDX3 was also shown to contribute to export of unspliced HIV mRNA, supporting HIV replication [74]. 

DDX1 is another stress granule resident protein [12] and has been found to inhibit or facilitate diverse viral infections including HIV, foot and mouth disease virus (FMDV), and transmissible gastroenteritis virus [18,71,75,76]. DDX1 was identified in a two-hybrid screen for HIV-Rev interactors and was shown to modulate the localization of Rev and the splicing of HIV mRNA. [77]. DDX1 acts as a chaperone to remodel the structure of the Rev-responsive element (RRE) RNA sequence, promoting increased Rev binding [75,78] and acting as a cofactor for Rev oligomerization [79]. The RRE can adopt several distinct structural conformations with differing affinities for Rev and functional consequences for HIV replication [80]. Another yeast two-hybrid screen led to the discovery that DDX1 supports the replication of the coronavirus infectious bronchitis virus (IBV), relocalizing to viral replication centers and interacting with the viral nonstructural protein nsP14; the ability of nsP14 to interact with DDX1 is conserved in severe acute respiratory syndrome coronavirus (SARS-CoV) [81]. Moreover, in a yeast model of Tombus virus infection, Ded1 and Dbp2 enhance viral replication by unwinding 3′ secondary structure to promote plus-strand RNA synthesis [82]. DDX1 inhibits foot and mouth disease virus and increases IFNβ production in infected cells [76]. DDX1 acts as a coactivator of NF-κB-mediated transcription via interaction with RelA, a function which requires intact helicase activity [19].

These interactions highlight the ways that both viruses and hosts can use RNP interactions to promote or antagonize one another’s function (Figure 2). The ability of some viruses to use DEAD-box helicase remodeling functions on their own RNA suggest that there may be other settings in which DEAD-box-helicase remodeling of viral RNA structure is detrimental to replication.

## 4. Summary and Discussion

The anti-viral and pro-viral DEAD-box helicase relationships that have been described so far in the literature likely represent only the tip of the iceberg in terms of the multitude of roles that DEAD-box helicases play in innate immunity and the modulation of viral infection (Table 1). These relationships were discovered through diverse approaches and have revealed interactions of viral infection with many aspects of RNA biology. Several examples have been identified through mass spectrometry approaches, arising either from studies investigating binding of viral proteins to host factors [35,60,83,84] or to nucleic acids [21,69,85]. Interactome studies have provided additional evidence for the importance of DEAD-box helicases in HIV and other viral infections [77,79,84,85,86,87]. Biochemical studies of HIV-Rev, alone or Rev in combination with RRE RNA, have also identified positive roles for DDX3X, DDX5, DDX17, and DDX21 in HIV infection, although some of these interactions could be indirect [84]. Other DEAD-box helicases have been found genetically by screening to identify genes that affect infection [41,88]. The genetic screening of DEAD-box helicases in poxvirus infection revealed both positive and negative regulation of infection by DEAD-box helicases [88]. And still other studies have focused on changes in gene expression that indicate either cellular innate immune responses or a viral manipulation of the host cell environment [31,89,90]. DDX3 was found to be upregulated in response to HIV-Tat expression, and contributes to the export of unspliced HIV mRNA, supporting HIV replication [74]. Microarray studies of HIV latency and reactivation found that DDX18 and DDX39 are upregulated during HIV latency and early reactivation and that the expression of additional DEAD-box helicases DDX10, DDX21, DDX23, and DDX52 is induced immediately following reactivation [89]. The biochemical characterization of these interactions provides crucial mechanistic insight into how these proteins impact infection. Confirming and defining protein–protein interactions with methods such as coimmunoprecipitation and defining regions of RNA binding by CLIP-seq have elucidated the mechanism of action of some of these proteins during viral infection. 

Although much remains to be defined about the antiviral roles of the DEAD-box helicase family, some broad themes have emerged. First, the functions of DEAD-box helicases in viral infection appear to often, but not always, rely on direct interactions between the host DEAD-box protein and viral RNAs. Some DEAD/DEAH-box helicases, such as the RLRs RIG-I and MDA-5, are specialized innate immune sensors, and others, such as DDX3X, have cellular roles but also contribute to innate immune signaling pathways. Still others bind viral RNA as antiviral effectors, whether induced by interferon signaling in the case of DDX60L or independently of known innate immune pathways, as is the case for DDX17 and DDX56. Continuing to biochemically define the RNA features recognized by antiviral helicases and the ways those RNA structures may be altered by helicase activity could help us identify functionally-important RNA structures in viral replication. Additionally, in recent years, the application of RNA probing technologies such as SHAPE to viral RNAs is beginning to enable detailed analysis of secondary and tertiary structures that may be functionally relevant [30,91,92]. A comparison of SHAPE data to DEAD-box helicase binding sites may enhance our understanding of structural motifs that stimulate innate immunity.

Second, DEAD-box helicase/virus interactions are highly context-dependent: different viruses are affected in different ways by the same DEAD-box helicases, and although RNA binding is important in many cases, there also exist examples where helicase function is dispensable for the antiviral activities that have been characterized. Detailed mechanistic studies naturally lag far behind the high-throughput genomic and proteomic identification of virus-host interactions, but a deeper investigation of the virus and host determinants underlying antiviral DEAD-box helicase function may illuminate key characteristics governing the outcome of a virus-helicase interaction.

Third, our understanding of the antiviral function of DEAD-box helicases is limited by a lack of information about their cellular roles. DEAD-box helicases are essential for many aspects of RNA biogenesis, but in many cases the details of their roles in these pathways have yet to be fully elucidated, and specific targets, activities, and cofactors are not well defined. Our limited knowledge of DEAD-box helicase function in both contexts hampers our ability to understand determinants governing RNA substrate selection and helicase activity and certainly our ability to predict whether or how they might interact with viral infection. We speculate that, in many cases, the viral substrates of these proteins might resemble their endogenous targets, whether through commonalities in target RNA secondary structure or in interactions with the variable N- and C-terminal domains that disrupt or coopt their cellular roles. As further examples and details of DEAD-box helicases in viral infection are characterized, complementary studies of their normal cellular functions will be necessary to determine how their interactions with viral infection resemble or differ from their normal biology. Ultimately, a better understanding of the virus–host interactions that affect the outcome of infection could lead to better antiviral therapeutics. Virus research has a long history of providing insight into cellular function, and these mechanisms will also teach us about cellular RNA regulation in broader contexts. This is an exciting time to explore the interface between viruses and cellular helicases.

## Figures and Tables

**Table 1:** DEAD-box helicase relationships with viral infections. Interactions are categorized as pro-viral if they support viral replication, and they are categorized as anti-viral if they counteract viral infection either directly or by activating innate immune signaling. Viral countermeasures are listed if a viral component disrupts an otherwise antiviral interaction. Other categories of interactions including gene expression changes are listed in the otherwise implicated column.

Gene	Pro-Viral	Anti-Viral	Viral Countermeasures	Otherwise Implicated
DDX1	IBV [81]	dsRNA sensing (with DDX21 and DHX36) [21] Transmissible gastroenteritis virus [18] NF-κB signaling [19]		
DDX3X	Arenavirus [38] JEV [47] HIV [74,84]	IFN-β induction (with TBK1) [26] Innate immune signaling [27,28] HBV [36,93] Myxoma virus [88]	VACV K7 [34] HBV polymerase [35] HCV 3′UTR [37]	
DDX5	JEV [47] HIV [84]	Myxoma virus [88]		
DDX6	Negative regulation of ISG induction [66]	RVFV, LACV [40] IVB [65]	Flaviviruses: sequestered by sfRNA [67,68]	
DDX10				HIV [89]
DDX17	HIV [84,94]	RVFV [41] Cofactor for ZAP [43] Tombusviruses [45]		
DDX18				HIV [89]
DDX21	HIV [84]	dsRNA sensing (with DDX1 and DHX36) [21] DENV [20] IVA [60] BDV [59]	DENV ns2B/3 [20]	IVA [22] HIV [89]
DDX23		dsRNA binding; VSV [48]		HIV [89]
DDX24	Attenuates RLR signaling [33]	RVFV [41]		
DDX39	IVA [95]			HIV [89]
DDX41		dsDNA sensing [25]		
DDX52		Myxoma virus [88]		HIV [89]
DDX56	WNV [54,55,56] HIV-1 [57,86]	RVFV [41]		
DDX60		VSV [31] HCV [32]		
DDX60L		HCV [29]		
